# Supplementary material for: Developmental Genetic Basis of Hoxd9 Homeobox Domain Deletion in Pampus argenteus Pelvic Fin Deficiency
Source: Int J Mol Sci. 2023 Jul 21;24(14):11769. doi: 10.3390/ijms241411769 (PMC10380636; doi:10.3390/ijms241411769)
Supplement: Supplementary file 1 [file ijms-24-11769-s001.zip › ijms-2453004-supplementary.pdf]

Supplementary data

The following are the Supplementary data to this article:

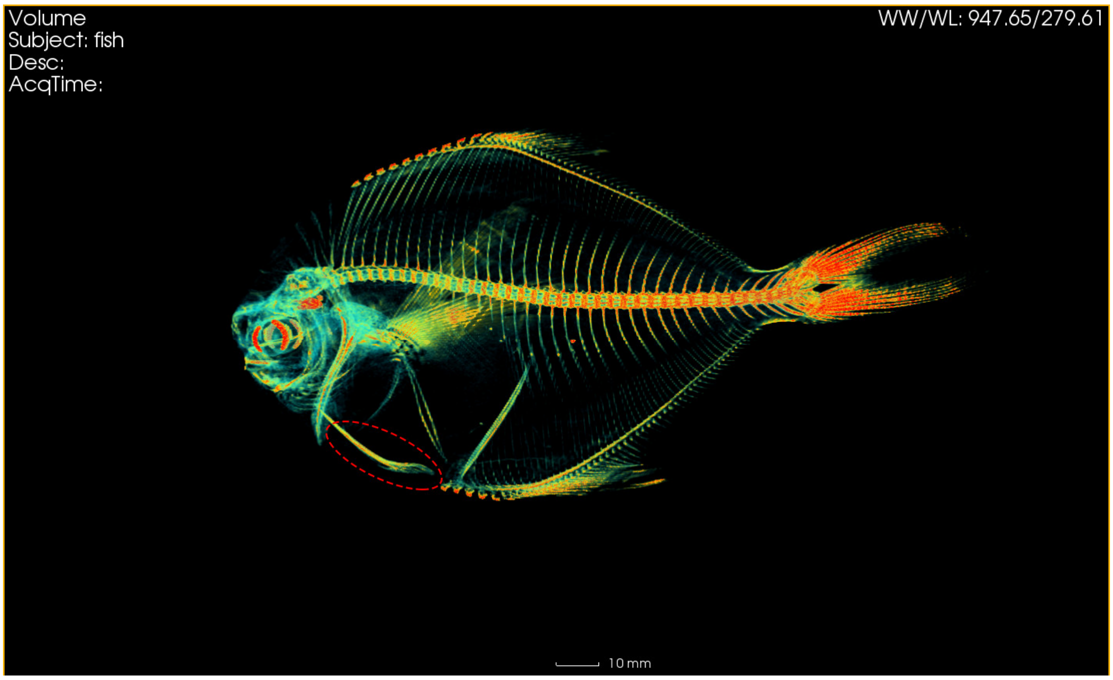

Supplementary Figure S1. Bone frame of *P. argenteus* by micro CT scanning. The red circle indicates the two girdle bones specialized from the hypothetical pelvic fin formation area of *P. argenteus*, which are thin in the front and thick in the back, with the front end connected with the cleithrum and the rear end at the front end of the cloaca (Unpublished).

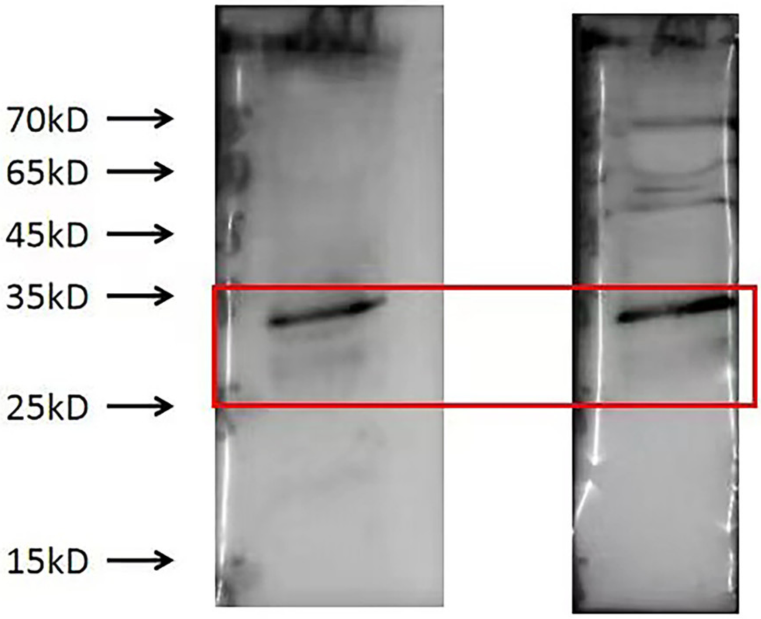

Supplementary Figure S2. Hoxd9a antibody test chart of *P. argenteus*. The protein molecular weight of *P. argenteus* Hoxd9 is 22.3 kDa, the dilution ratio is 1:100, and bands between 25 and 35 kDa are observed.

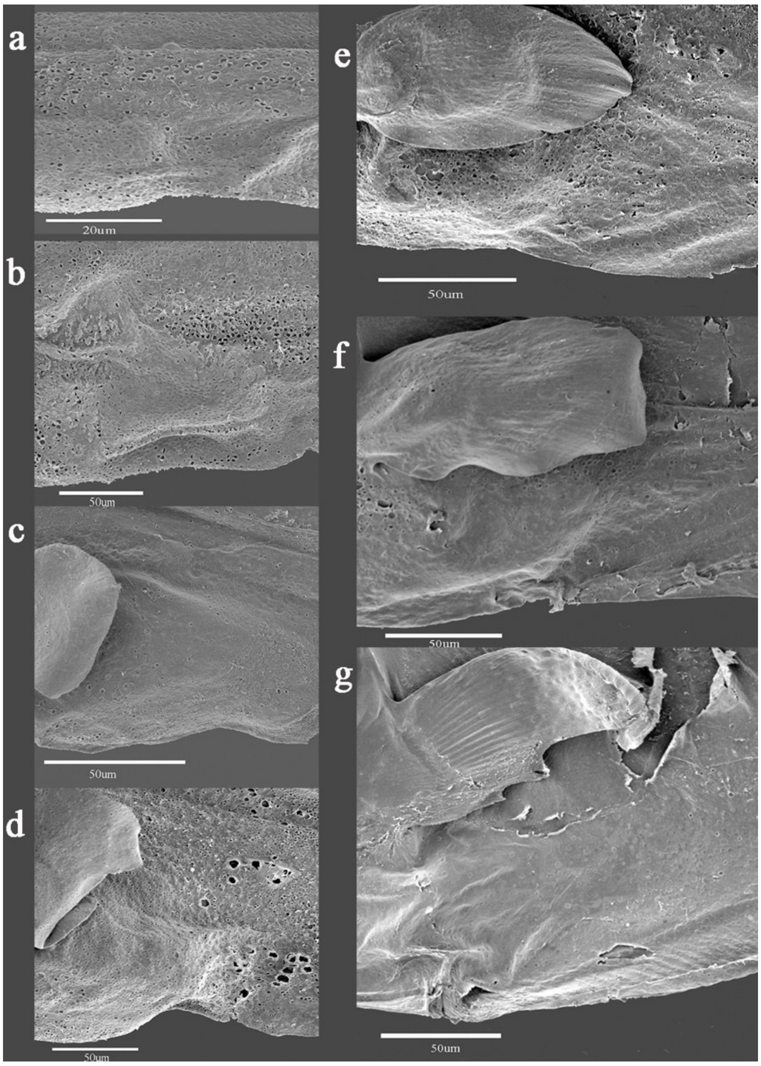

Supplementary Figure S3. Scanning electron microscope observation of *P. argenteus* larvae. a: 1 days larvae, SL 500 um; b: 3 days larvae, SL 500 um; c: 7 days larvae, SL 500 um; d: 13 days larvae, SL 1.00 mm; e: 16 days juveniles, SL 1.00 mm; f: 19 days juvenils, SL 1.00 mm; g: 25 days juveniles, SL 2.00 mm. a: Scale Bar=20 um; b-g: Scale Bar=50 um.

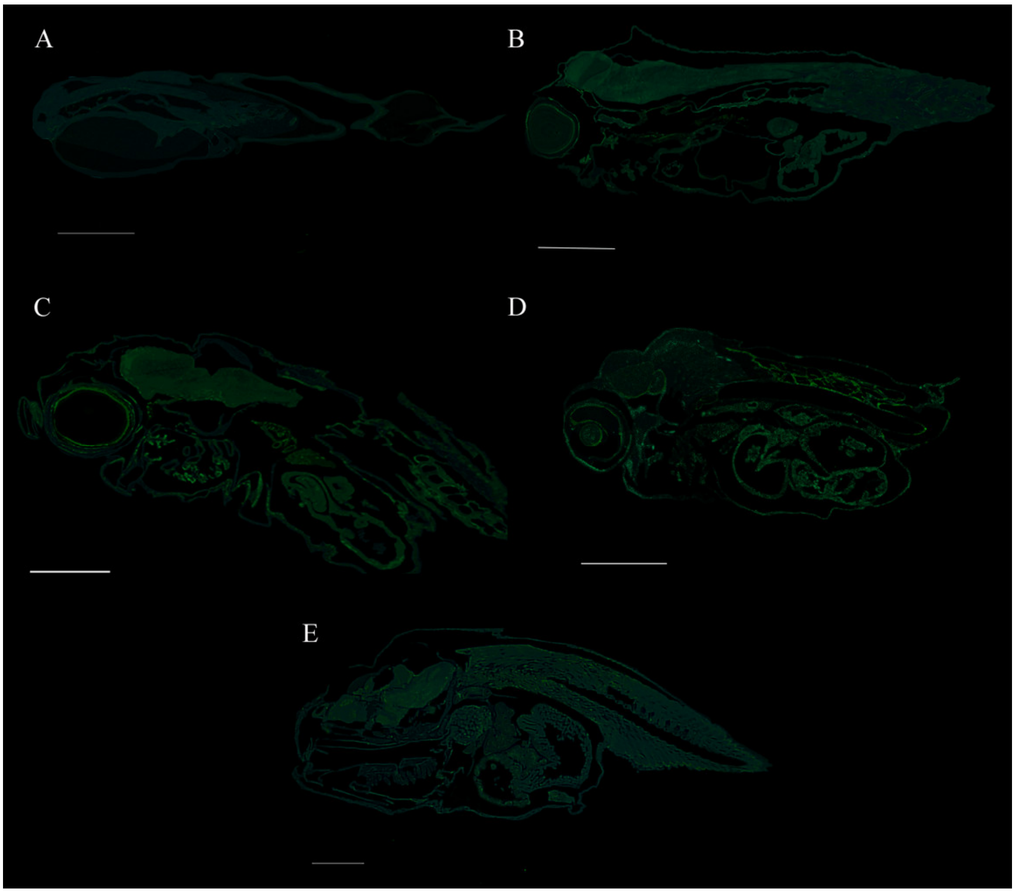

Supplementary Figure S4. Fluorescence in situ hybridization of the *Hoxd9* gene in *P. argenteus* at different developmental stages in the control group. A: 1-day-old larvae, b: 7-day-old larvae, c: 13-day-old larvae, d: 16-day-old juvenile and e: 19-day-old juvenile. Bars=500 um.

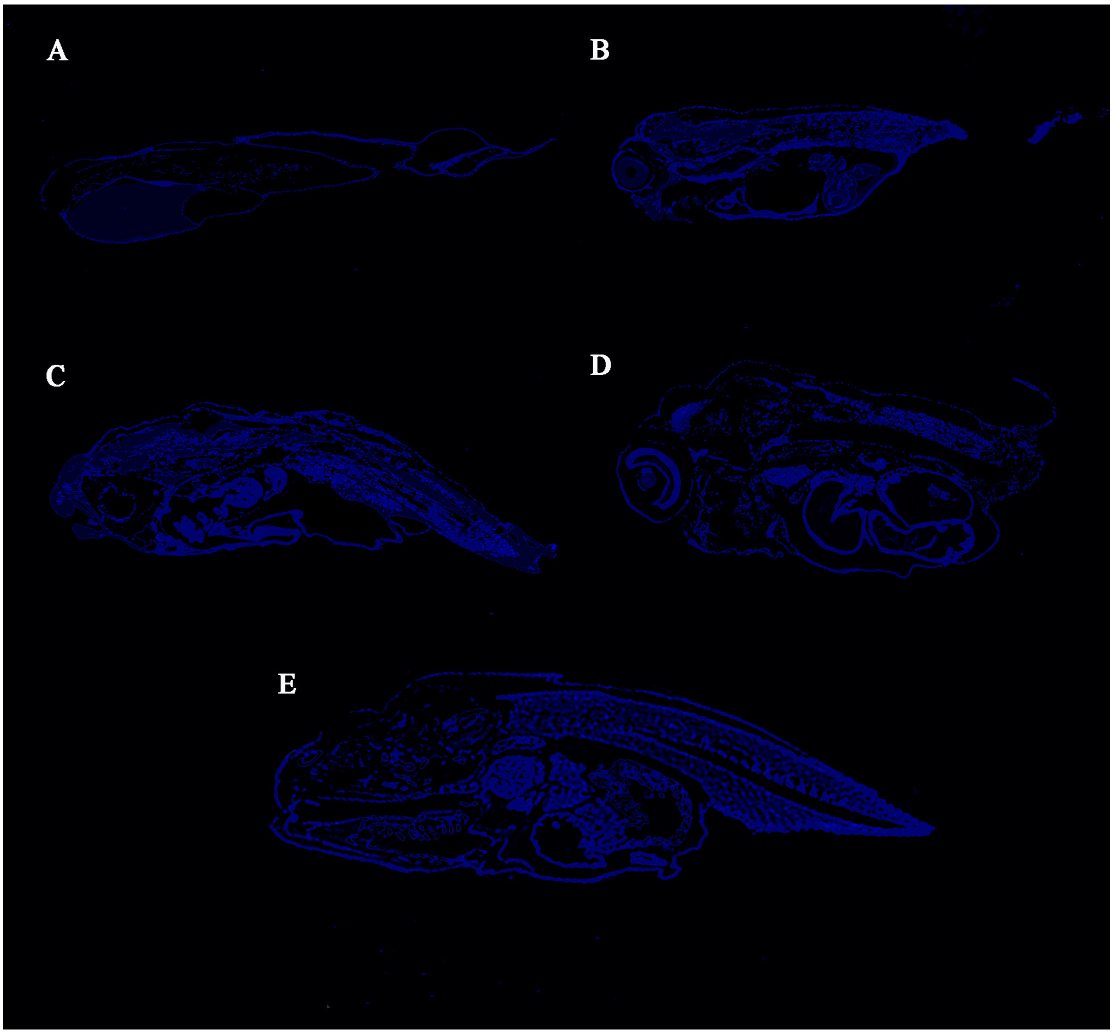

Supplementary Figure S5. Immunofluorescence of the HOXD9 protein in *P. argenteus* at different developmental stages in the control group. A: 1-day-old larvae, b: 7-day-old larvae, c: 13-day-old larvae, d: 16-day-old juvenile and e: 19-day-old juvenile. Bars=1000 um.
